# PReS-FINAL-2364: Status epilepticus as an initial manifestation of central nervous system small vessel vasculitis is related to unfavourable neurological outcome in paediatric patients

**DOI:** 10.1186/1546-0096-11-S2-P354

**Published:** 2013-12-05

**Authors:** V Krakovská, M Twilt, S Sheikh, P Doležalová, SM Benseler

**Affiliations:** 1 Division of Rheumatology, Department of Paediatrics, The Hospital for Sick Children University of Toronto, Toronto, Canada; 2 Rheumatology Unit, Department of paediatrics and adolescent medicine, First Faculty of Medicine, Charles University in Prague and General University Hospital in Prague, Prague, Czech Republic; 3 Division of Rheumatology, Birmingham Children's hospital, Birmingham, UK

## Introduction

Childhood primary angiitis of the central nervous system (cPACNS) is an inflammatory disease involving small and medium-large size brain vessels with variable clinical presentation. Clinical symptoms range from headache, cognitive impairment, hemiplegia, language deficit to seizures and status epilepticus. However, it remains unclear if the type of initial manifestation/symptoms predicts neurological outcome in cPACNS.

## Objectives

To assess the relationship between the clinical manifestation at disease onset and medium to long-term neurological outcome of cPACNS.

## Methods

All consecutive children (< 18 years) diagnosed with small vessel CNS vasculitis, according Calabrese criteria and consented to the BrainWorks network at the Hospital for Sick Children in Toronto in the last 10 years (between January 2002 and November 2012) with a minimum follow-up of 4 months were included in this study. All prospective clinical, laboratory, imaging and histopathology data were captured in the BrainWorks database. Patients were divided in three subgroups according to the seizure type at clinical presentation: status epilepticus, seizures and patients without status epilepticus or seizures but with other neuropsychiatric abnormalities. Outcome assessments were evaluated by validated and standardized paediatric stroke outcome measure (PSOM) and Physician global assessment (PGA), which estimated on a visual analogue scale disease activity (PGA-DA), reversible changes (PGA-RC) and permanent damage (PGA-PD).

## Results

The study cohort included 39 patients (25 girls, 64%) with biopsy confirmed small vessel cPACNS. Mean age at disease onset was 10.1 ± 4.1 years with a follow-up of 4 month up to 2 years. Status epilepticus was an initial cPACNS manifestation in 9 patients (23%, 3 girls, 33%). Seizures were present in 17 patients (44%, 12 girls, 71%) and 13 patients (33%, 10 girls, 77%) had no seizures or status but had other neurological symptoms including a decrease of consciousness, focal neurological deficit or headache. Patients with status epilepticus had the worst PSOM (p = 0.001) and PGA-RC a PGA-RD (p = 0.002) at disease onset. This subgroup also shows an increase in PGA-DA (p = 0.12) at follow-up. Patients presenting with status epilepticus tended to be more likely boys (6 patients, 66%) as compared (p = 0.084) to the other 2 groups. There was no significant difference in time to diagnosis among the groups (p = 0.19)

## Conclusion

Children with small vessel cPACNS presenting with status epilepticus as a first disease manifestation have poorer medium-long term clinical outcome as compared to other cPACNS patients. Interestingly, patients with status epilepticus tended to be more likely boys.

## Disclosure of interest

None declared.

